# Association between protein-energy wasting and cognitive impairment in maintenance hemodialysis patients

**DOI:** 10.3389/fnut.2026.1825670

**Published:** 2026-04-29

**Authors:** Xiaobao Wei, Ting Zhang, Meng Sun, Xinyu Xiang, Yue Qin, Meichen Zhang, Liyuan Zhang

**Affiliations:** 1 Department of Nephrology, Lianyungang Clinical College of Xuzhou Medical University, Lianyungang, Jiangsu, China; 2 Department of Nephrology, The First People’s Hospital of Lianyungang, Lianyungang, Jiangsu, China; 3 Department of Nephrology, Lianyungang Clinical College of Nanjing Medical University, Lianyungang, Jiangsu, China

**Keywords:** cognitive impairment, maintenance hemodialysis, malnutrition-inflammation score, mid-arm muscle circumference, protein-energy wasting

## Abstract

**Objective:**

This study aimed to investigate the association between protein-energy wasting (PEW) and cognitive impairment (CI) in patients undergoing maintenance hemodialysis (MHD).

**Methods:**

In this single-center cross-sectional study, 86 patients receiving MHD were enrolled. Nutritional status was assessed using anthropometric measurements, including mid-arm circumference (MAC) and mid-arm muscle circumference (MAMC), as well as the Malnutrition-Inflammation Score (MIS). Cognitive function was evaluated with the Montreal Cognitive Assessment (MoCA), with patients classified into CI (n = 50) and non-CI (n = 36) groups based on education-adjusted scores. Group comparisons, Spearman correlation analysis, multivariable logistic regression, and receiver operating characteristic (ROC) curve analysis were performed.

**Results:**

Cognitive impairment was present in 58.1% of the patients. Compared with the non-CI group, patients with CI had a higher Malnutrition–Inflammation Score and lower MAMC. Multivariable logistic regression analysis showed that lower MAMC (adjusted OR 0.571, 95% CI 0.400–0.816, *p* = 0.002) and higher MIS (adjusted OR 1.249, 95% CI 1.006–1.550, *p* = 0.044) were independently associated with cognitive impairment. ROC analysis demonstrated that a multivariable model combining age, years of education, dialysis vintage, MAMC, and MIS exhibited excellent discriminative ability (AUC = 0.942, 95% CI 0.898–0.987).

**Conclusion:**

Lower MAMC and higher MIS were independently associated with cognitive impairment in patients undergoing maintenance hemodialysis. These two indicators (MAMC and MIS) showed stronger and more consistent associations with cognitive impairment than single biochemical markers. Given the cross-sectional design of the study, the temporal direction of the association cannot be determined. Prospective studies are warranted to clarify whether muscle loss precedes cognitive decline or occurs as a consequence of it, and to evaluate whether preserving muscle mass can protect cognitive function in this population.

## 1 Introduction

End-stage renal disease (ESRD) patients rely on renal replacement therapy to sustain life, with maintenance hemodialysis (MHD) serving as the primary treatment. Studies have confirmed that the prevalence of CI among MHD patients is high, typically ranging from 40% to 80% (1, 2). Patients also carry a substantially increased risk of developing cognitive impairment after initiating hemodialysis (3). Cognitive impairment not only reduces treatment adherence, increases hospitalization risk, and may lead to discontinuation of dialysis, but is also recognized as an independent predictor of all-cause mortality in MHD patients (4, 5). However, effective approaches to identify, prevent, and manage cognitive impairment in dialysis patients remain lacking.

Malnutrition is also a common issue among hemodialysis patients. The prevalence of malnutrition in patients with hemodialysis treatment has been reported as up to 91%, but this may vary depending on the method with which it is diagnosed. (6) Some research has observed correlations between certain nutritional indicators and changes in specific cognitive functions (7, 8). Furthermore, many studies have found that CI is more common among malnourished hemodialysis patients (9). However, routine dietary assessments often fail to capture the complex metabolic abnormalities associated with chronic kidney disease. Protein-energy wasting (PEW), characterized by biochemical abnormalities, weight loss, muscle depletion, and insufficient caloric intake, provides a more comprehensive metabolic profile (10, 11).

The diagnosis of PEW requires meeting relevant criteria across various domains, with muscle mass measured by markers such as mid-arm muscle circumference (MAMC) being a critical component (11). Although PEW is prevalent (23%–76%) in dialysis patients (12), its relationship to CI has not been adequately explored. It remains unclear whether individual components of PEW, including muscle mass or inflammation-related scores, are independently associated with cognitive impairment.

This study aimed to investigate the relationships between PEW-related markers and CI in patients undergoing MHD, with the objective of offering new insights for the identification and comprehensive management of CI in this demographic.

## 2 Materials and methods

### 2.1 Study design and participants

We consecutively enrolled 86 MHD patients at a university-affiliated hemodialysis center between August 2023 and December 2024. Inclusion criteria were: (1) age ≥18 years; (2) end-stage renal disease receiving maintenance hemodialysis for at least 6 months, with a regular regimen of three sessions per week, each lasting 4 h; (3) ability to provide informed consent. Exclusion criteria included: (1) a history of stroke or intracranial hemorrhage; (2) diagnosed psychiatric or neurodegenerative disorders; (3) irregular hemodialysis attendance (defined as fewer than three sessions per week or missed sessions without medical justification); (4) blood transfusion or cognitive assessment within the previous 3 months; (5) severe sensory, motor, or communication impairments that precluded cognitive assessment; (6) presence of metallic implants or limb amputation; and (7) acute clinical instability, defined as hospitalization or severe acute illness within the 3 months prior to enrollment. All participants provided written informed consent, and the study was approved by the Institutional Review Board of the hospital (Approval No. KY-20241219001-01).

### 2.2 Clinical and laboratory assessments

Demographic and clinical data were collected using standardized questionnaires. The demographic data included sex, age, comorbidities, years of education, fatigue related to physical labor (or occupational fatigue), alcohol consumption, and smoking history. For laboratory data, fasting blood samples were collected before a midweek hemodialysis session. Routine laboratory tests were then performed, including complete blood count, serum albumin, prealbumin, high-sensitivity C-reactive protein (hs-CRP), calcium, phosphorus, magnesium, lipid profile, renal function, uric acid, urea clearance index and other relevant indicators.

### 2.3 Cognitive function evaluation

Global cognitive function was assessed using the Montreal Cognitive Assessment (MoCA). To correct for educational level, one additional point was added for participants with ≤12 years of education. CI was defined as an adjusted MoCA score <26 (13, 14). All assessments were performed by trained nephrologists in a quiet room prior to a midweek hemodialysis session. Executive function and attention were further evaluated using the Trail Making Test (TMT) Parts A and B. The TMT is a neuropsychological test used to assess executive function, attention, and visuomotor speed. TMT-A primarily measures visual scanning and processing speed, while TMT-B additionally evaluates cognitive flexibility and task-switching ability. Completion time (in seconds) and the number of errors were recorded.

### 2.4 Nutritional status assessment

Anthropometric measurements were performed by a single trained operator after a midweek hemodialysis session on the arm without arteriovenous fistula. Mid-arm circumference (MAC) and triceps skinfold thickness (TSF) were measured in triplicate, and the average values were used for calculations. MAMC was derived using the formula: MAMC (cm) = MAC (cm) − *π* × TSF (cm).

Dietary intake was assessed by trained staff using a three-day food record, including one hemodialysis day and two non-dialysis days. Average daily protein intake (DPI; g/kg ideal body weight/day) and daily energy intake (DEI; kJ/kg ideal body weight/day) were calculated. For reference, 1 kcal equals 4.186 kJ.

PEW was diagnosed based on the 2008 International Society of Renal Nutrition and Metabolism (ISRNM) criteria (10). This required abnormalities in at least three of the four main categories: (1) Biochemical indicators: albumin <38 g/L, prealbumin <300 mg/L, cholesterol <2.59 mmol/L. (2) Body weight indicators: body mass index (BMI) < 23 kg/m^2^, unintentional weight loss (>5% within 3 months or >10% within 6 months), body fat mass <10%. (3) Muscle indicators: muscle mass loss (>5% within 3 months or >10% within 6 months), MAMC reduced by >10% compared to a standard population. (4) Dietary intake: DPI < 0.8 g/kg/day for more than 2 months, DEI < 105 kJ/kg/day for more than 2 months. Additional nutritional evaluations included the 7-point Subjective Global Assessment (SGA), the Malnutrition-Inflammation Score (MIS), and the Nutritional Risk Screening 2002 (NRS-2002). The 7-point SGA evaluates nutritional status based on medical history (weight change, dietary intake, gastrointestinal symptoms, and functional capacity) and physical examination (subcutaneous fat loss and muscle wasting). Scores range from 1 to 7, with higher scores indicating better nutritional status. Patients are classified as well-nourished (SGA A), mildly to moderately malnourished (SGA B), or severely malnourished (SGA C).

### 2.5 Sample size

The sample size was determined pragmatically by consecutive enrollment of all eligible patients at our hemodialysis center during the study period (August 2023 to December 2024). A total of 86 patients were included, providing 50 events of cognitive impairment. The statistical adequacy of the final multivariable model was evaluated using the “events per variable” (EPV) guideline proposed by Peduzzi et al. (15). The final parsimonious multivariable model included 5 variables (age, years of education, dialysis vintage, MAMC, and MIS), yielding an EPV of 10.0. This ratio meets the conventional threshold of 10 recommended by Peduzzi et al. to ensure model stability. Additionally, all variance inflation factors (VIF) remained below 5, indicating no substantial multicollinearity. Furthermore, the model demonstrated excellent discriminative performance (AUC = 0.942, 95% CI 0.898–0.987), supporting the robustness of the findings in this exploratory study.

### 2.6 Statistical analysis

All statistical analyses were performed using IBM SPSS Statistics version 27.0. Continuous variables with normal distribution are presented as mean ± SD and were compared using the Student’s t-test, while non-normally distributed continuous variables are presented as median (interquartile range) and were compared using the Mann–Whitney U test. Categorical variables are presented as number (percentage) and were compared using the *χ*^2^ test or Fisher’s exact test, as appropriate. Spearman’s rank correlation analysis was used to examine the correlations between the MoCA total score, cognitive subdomain scores, and nutritional indicators (including PEW and related parameters).

Variables with univariable *p* < 0.05 were considered candidates for multivariable logistic regression using the Enter method. To avoid structural multicollinearity and clinical conceptual overlap, the composite PEW diagnosis and serum albumin were excluded *a priori* (as albumin is a scored component of the Malnutrition-Inflammation Score). VIF were calculated to assess multicollinearity in the final model. Receiver operating characteristic (ROC) curve analysis was performed to evaluate the discriminative performance of the identified variables. All tests were two-tailed, and a *p*-value < 0.05 was considered statistically significant.

## 3 Results

### 3.1 Baseline characteristics and prevalence of CI

**Figure 1:**
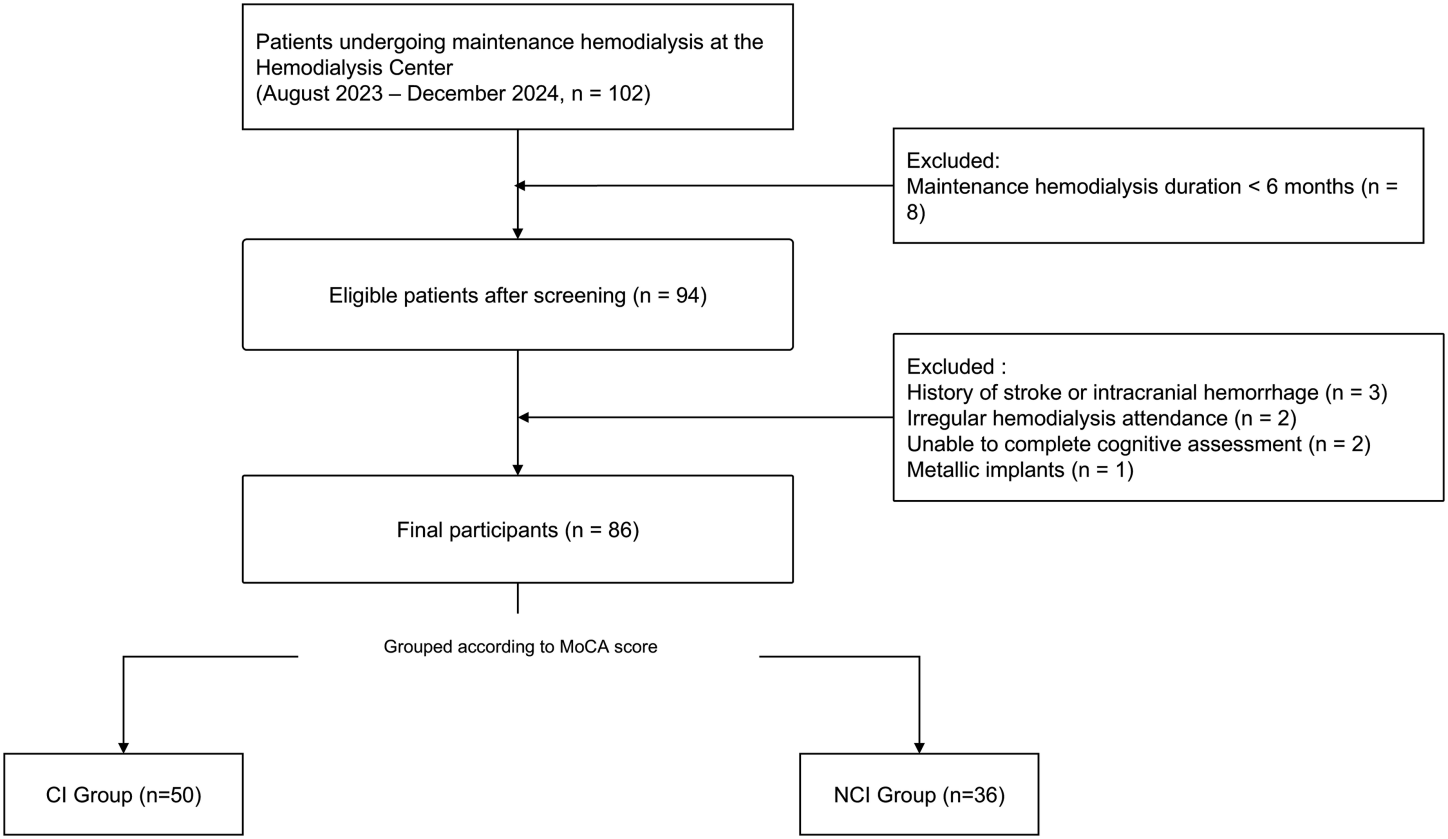
Study flow diagram. Flow diagram showing patient screening, exclusion, and final inclusion in the cross-sectional study. Between August 2023 and December 2024, 102 patients receiving maintenance hemodialysis were screened. After exclusions, 86 patients were included in the final analysis.

Between August 2023 and December 2024, 102 patients undergoing maintenance hemodialysis at our center were consecutively screened. Sixteen patients were excluded because their dialysis duration was less than 6 months (*n* = 8), they had a history of stroke or intracranial hemorrhage (*n* = 3), showed irregular hemodialysis attendance (*n* = 2), were unable to complete the cognitive assessment (*n* = 2), or had metallic implants (*n* = 1). The remaining 86 patients were included in this cross-sectional study (Figure 1).

Of the 86 patients undergoing maintenance hemodialysis, 50 (58.1%) had cognitive impairment according to an education-adjusted MoCA score < 26. Compared with patients without cognitive impairment, those with cognitive impairment had higher C-reactive protein levels (*p* = 0.045) and lower serum albumin levels (*p* = 0.032). No significant differences were observed in sex, hemoglobin, urea clearance index or lipid profiles (Table 1).

**Table 1:** Comparison of baseline characteristics between CI and NCI groups.

Variables	CI group (*n* = 50)	NCI group (*n* = 36)	*χ*^2^/*Z*/*t*	*p*-value
Age, years	58.00 (44.00, 61.00)	42.50 (37.00, 49.75)	3.688	<0.001
Male, *n* (%)	28 (56.0)	25 (69.4)	1.600	0.206
Hypertension, *n* (%)	44 (88.0)	30 (83.3)	0.380	0.538
Diabetes, *n* (%)	6 (12.0)	8 (22.2)	1.605	0.205
Dialysis vintage, years	6.50 (4.50, 11.00)	2.25 (1.00, 4.50)	4.716	<0.001
Education, years	9.00 (6.75, 12.00)	12.00 (10.00, 15.00)	−3.656	<0.001
Work-related fatigue, *n* (%)	17 (34.0)	12 (33.3)	0.004	0.949
Smoker, *n* (%)	16 (32.0)	8 (22.2)	0.995	0.319
Drinker, *n* (%)	6 (12.0)	2 (5.6)	0.408	0.523
Albumin, g/L	37.60 (34.73, 39.80)	39.80 (37.73, 41.90)	−2.141	0.032
hs-CRP, mg/L	5.94 (2.08, 10.07)	4.49 (2.17, 5.75)	2.009	0.045
Prealbumin, g/L	0.30 (0.28, 0.30)	0.30 (0.30, 0.30)	−1.716	0.086
Hemoglobin, g/L	106.44 ± 15.70	108.69 ± 14.74	0.674	0.502
White blood cell, ×10^9^/L	5.37 ± 1.18	5.84 ± 1.49	1.607	0.112
Neutrophil, ×10^9^/L	3.42 (2.88, 4.32)	3.85 (3.01, 5.41)	−1.856	0.063
Lymphocyte, ×10^9^/L	1.24 ± 0.38	1.08 ± 0.34	−1.990	0.050
Platelet, ×10^9^/L	176.50 (156.50, 209.50)	192.50 (157.25, 223.25)	−1.274	0.203
Parathyroid hormone, pg./mL	235.50 (52.64, 405.00)	170.50 (42.75, 331.75)	0.657	0.511
Potassium, mmol/L	4.57 ± 0.49	4.45 ± 0.52	−1.087	0.280
Calcium, mmol/L	2.17 ± 0.21	2.18 ± 0.17	0.345	0.731
Phosphorus, mmol/L	1.85 ± 0.43	1.80 ± 0.41	−0.597	0.552
Magnesium, mmol/L	0.95 (0.84, 1.06)	0.92 (0.87, 1.03)	−0.158	0.875
25-Hydroxyvitamin D, ng/mL	12.71 (10.03, 14.90)	12.91 (10.59, 17.09)	−0.906	0.365
Serum creatinine, μmol/L	994.70 (777.02, 1,133.98)	990.10 (665.95, 1,131.20)	0.105	0.916
Urea nitrogen, mmol/L	23.30 (18.93, 26.48)	23.50 (18.53, 32.25)	−0.709	0.478
Kt/V	1.31 ± 0.20	1.33 ± 0.17	0.538	0.592
Serum iron, μmol/L	9.40 (8.16, 14.39)	11.85 (8.18, 14.40)	−0.911	0.363
Total cholesterol, mmol/L	3.80 (3.03, 4.13)	3.80 (3.13, 4.12)	−0.552	0.581
Triglycerides, mmol/L	1.23 (0.83, 2.01)	1.42 (0.93, 2.17)	−1.261	0.207
LDL-C, mmol/L	2.26 (1.90, 2.53)	2.54 (1.97, 3.00)	−1.825	0.068
Uric Acid, μmol/L	383.59 ± 118.54	389.59 ± 119.32	0.231	0.818

CI, cognitive impairment; NCI, non-cognitive impairment; hs-CRP, high-sensitivity C-reactive protein; LDL-C, low-density lipoprotein cholesterol; Kt/V, urea clearance index (single-session spKt/V).

### 3.2 Comparison of nutritional status and PEW-related indicators

Patients with CI had a significantly higher prevalence of PEW (36.0% vs. 11.1%, *p* = 0.009), lower mid-arm circumference and MAMC (both *p* < 0.001), and higher Malnutrition–Inflammation Score (median 6 vs. 3, *p* < 0.001) (Table 2).

**Table 2:** Comparison of nutritional status and PEW-related indicators between CI and NCI groups.

Variables	CI group (*n* = 50)	NCI group (*n* = 36)	*χ*^2^/*Z*/*t*	*p*-value
PEW, *n* (%)	18 (36.0)	4 (11.1)	6.811	0.009
Albumin <38 g/L, *n* (%)	28 (56.0)	11 (30.6)	5.385	0.020
Prealbumin <300 mg/L, *n* (%)	50 (100)	34 (94.4)	2.941	0.086
Total cholesterol <2.59 mmol/L, *n* (%)	7 (14.0)	2 (5.6)	1.605	0.205
BMI (kg/m^2^)	22.16 (19.85, 27.55)	24.72 (22.34, 27.43)	−1.046	0.296
Unintentional weight loss, *n* (%)	7 (14.0)	2 (5.6)	0.819	0.365
Body fat percentage <10%, *n* (%)	2 (4.0)	1 (2.8)	0.000	1.000
Muscle mass loss, *n* (%)	9 (18.0)	3 (8.3)	1.629	0.202
MAC, cm	27.52 ± 2.41	29.55 ± 3.03	3.439	<0.001
MAMC, cm	22.65 ± 2.25	24.55 ± 2.32	3.815	<0.001
DPI (g/kg/day)	1.16 (1.08, 1.27)	1.08 (1.02, 1.24)	1.480	0.139
DPI < 0.8 g/kg/day, *n* (%)	0 (0)	1 (2.8)	1.415	0.234
DEI (kJ/kg/day)	106.4 ± 6.7	104.1 ± 11.8	−1.059	0.295
DEI < 105 kJ/kg/day, *n* (%)	23 (46.0)	20 (55.6)	0.757	0.384
MIS	6 (4, 9)	3 (2, 5)	4.209	<0.001
SGA, *n* (%)			0.177	0.674
Well nourished (A)	34 (68.0)	26 (72.2)		
Mild/moderately malnourished (B)	16 (32.0)	10 (27.8)		
NRS2002 score	0 (0, 0)	0 (0, 0)	0.954	0.340

CI, cognitive impairment; NCI, non-cognitive impairment; PEW, protein-energy wasting; MAC, mid-arm circumference; MAMC, mid-arm muscle circumference; DPI, dietary protein intake; DEI, dietary energy intake; MIS, Malnutrition–Inflammation Score; SGA, Subjective Global Assessment; NRS2002, Nutritional Risk Screening 2002.

### 3.3 Comparison of cognitive function

Patients with CI scored significantly lower than those without CI across all seven domains of the MoCA. They also performed worse on the TMT, with longer completion times for both TMT-A and TMT-B, more sequencing errors on TMT-B, and fewer correct connections (all *p* < 0.001; see Supplementary Table S1 for details).

### 3.4 Associations between cognitive function and nutritional parameters

**Figure 2:**
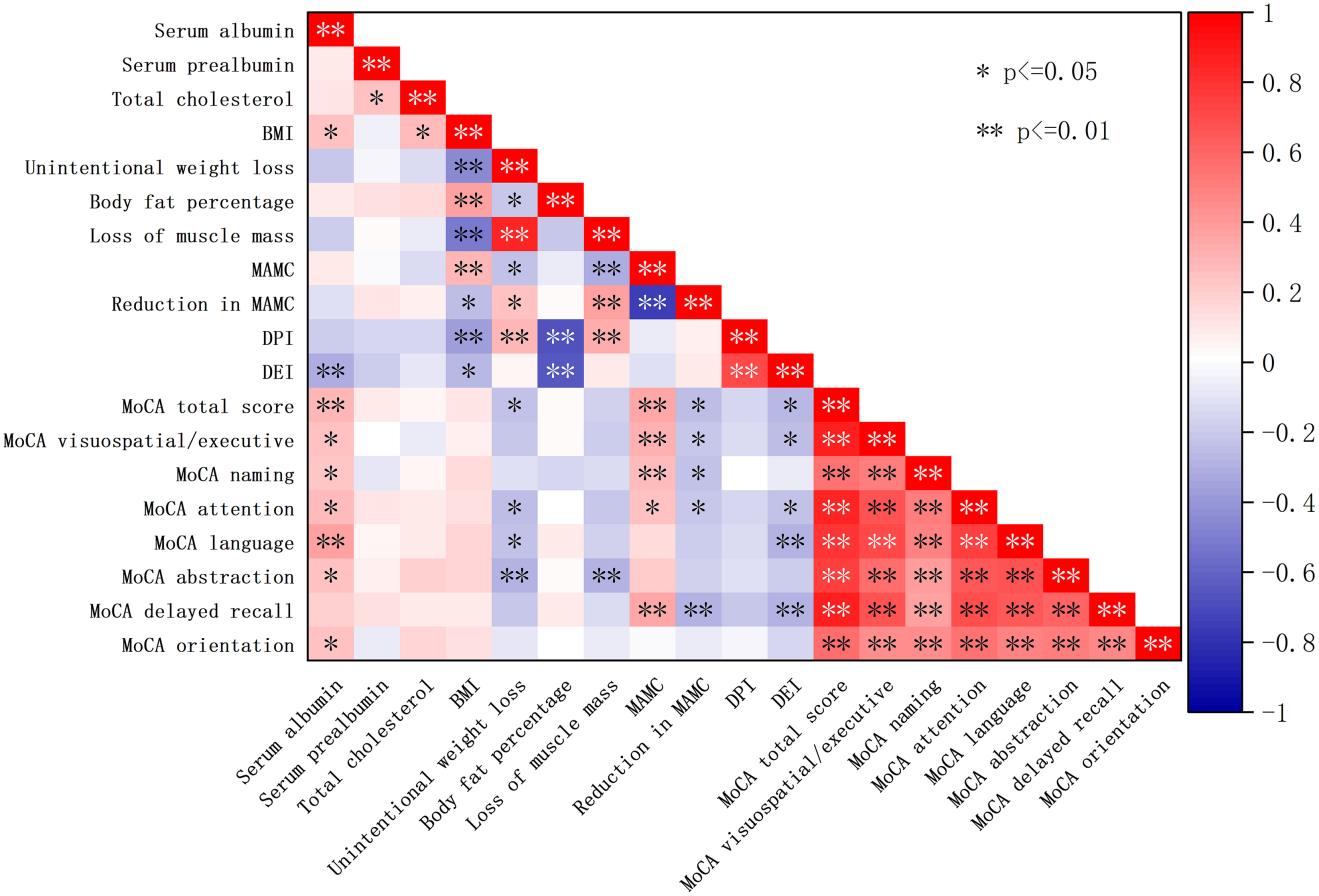
Associations between nutritional parameters and cognitive function. Spearman correlation heatmap showing the relationships between MoCA scores (total and subdomain scores) and PEW-related nutritional indicators. Red colors represent positive correlations and blue colors represent negative correlations, with color intensity indicating the strength of the correlation. Lower MAMC and higher MIS were associated with lower MoCA scores.

Spearman correlation analysis showed that PEW was negatively correlated with total MoCA score (r_s_ = −0.309, *p* = 0.004), whereas MAMC (r_s_ = 0.324, *p* = 0.003) and serum albumin (r_s_ = 0.281, *p* = 0.009) were positively correlated with MoCA score. Domain-specific correlations and subgroup comparisons are visualized in Figure 2.

### 3.5 Factors associated with cognitive impairment

In the multivariable logistic regression analysis using the Enter method, older age (adjusted OR 1.089, 95% CI 1.016–1.168, *p* = 0.017), longer dialysis vintage (adjusted OR 1.384, 95% CI 1.121–1.709, *p* = 0.002), fewer years of education (adjusted OR 0.736, 95% CI 0.587–0.923, *p* = 0.008), lower MAMC (adjusted OR 0.571, 95% CI 0.400–0.816, *p* = 0.002), and higher MIS (adjusted OR 1.249, 95% CI 1.006–1.550, *p* = 0.044) were independently associated with cognitive impairment in the final parsimonious model. The composite PEW diagnosis was not included due to substantial conceptual overlap with its key components. Serum albumin, despite its marginal univariable significance (*p* = 0.052), was excluded from the multivariable model to avoid structural multicollinearity, as it is a constituent component of the MIS. Furthermore, although hs-CRP was significant in the univariable analysis, it lost independent significance after multivariable adjustment (adjusted OR 1.133, 95% CI 0.929–1.381, *p* = 0.217). Consequently, this variable was not included in the final parsimonious model or the subsequent ROC analysis (see Table 3).

**Table 3:** Univariate and multivariable logistic regression analysis of factors associated with CI.

Variables	Univariate analysis		Multivariable analysis	
	OR (95% CI)	*p*-value	Adjusted OR (95% CI)	*p*-value
Age (per 1-year increase)	1.088 (1.040–1.139)	<0.001	1.089 (1.016–1.168)	0.017
Education (per 1-year increase)	0.788 (0.687–0.904)	0.001	0.736 (0.587–0.923)	0.008
Dialysis vintage (per 1-year increase)	1.393 (1.186–1.636)	<0.001	1.384 (1.121–1.709)	0.002
MIS (per 1-point increase)	1.378 (1.143–1.662)	<0.001	1.249 (1.006–1.550)	0.044
MAMC (per 1-cm increase)	0.699 (0.566–0.862)	0.001	0.571 (0.400–0.816)	0.002
hs-CRP (mg/L)	1.165 (1.027–1.320)	0.017	—	-—
Albumin (g/L)	0.902 (0.813–1.001)	0.052	—	—
PEW (yes vs. no)	4.500(1.370–14.778)	0.013	—	—

Multivariable logistic regression was performed using the Enter method. Variables with univariable *p* < 0.05 were entered as candidate variables. The final parsimonious model included only 5 variables: age, years of education, dialysis vintage, MAMC, and MIS (EPV = 10). The composite PEW diagnosis was excluded a priori due to conceptual overlap with its components. Serum albumin was excluded due to multicollinearity with MIS. hs-CRP was evaluated in the initial multivariable model but lost independent significance after adjustment (adjusted OR 1.133, 95% CI 0.929–1.381, *p* = 0.217) and was therefore not included in the final model. All variance inflation factors (VIF) were < 5.

CI, cognitive impairment; EPV, events per variable; hs-CRP, high-sensitivity C-reactive protein; MAMC, mid-arm muscle circumference; MIS, Malnutrition–Inflammation Score; OR, odds ratio; PEW, protein-energy wasting; VIF, variance inflation factor.

### 3.6 Cross-sectional discriminative ability of key risk factors

**Figure 3:**
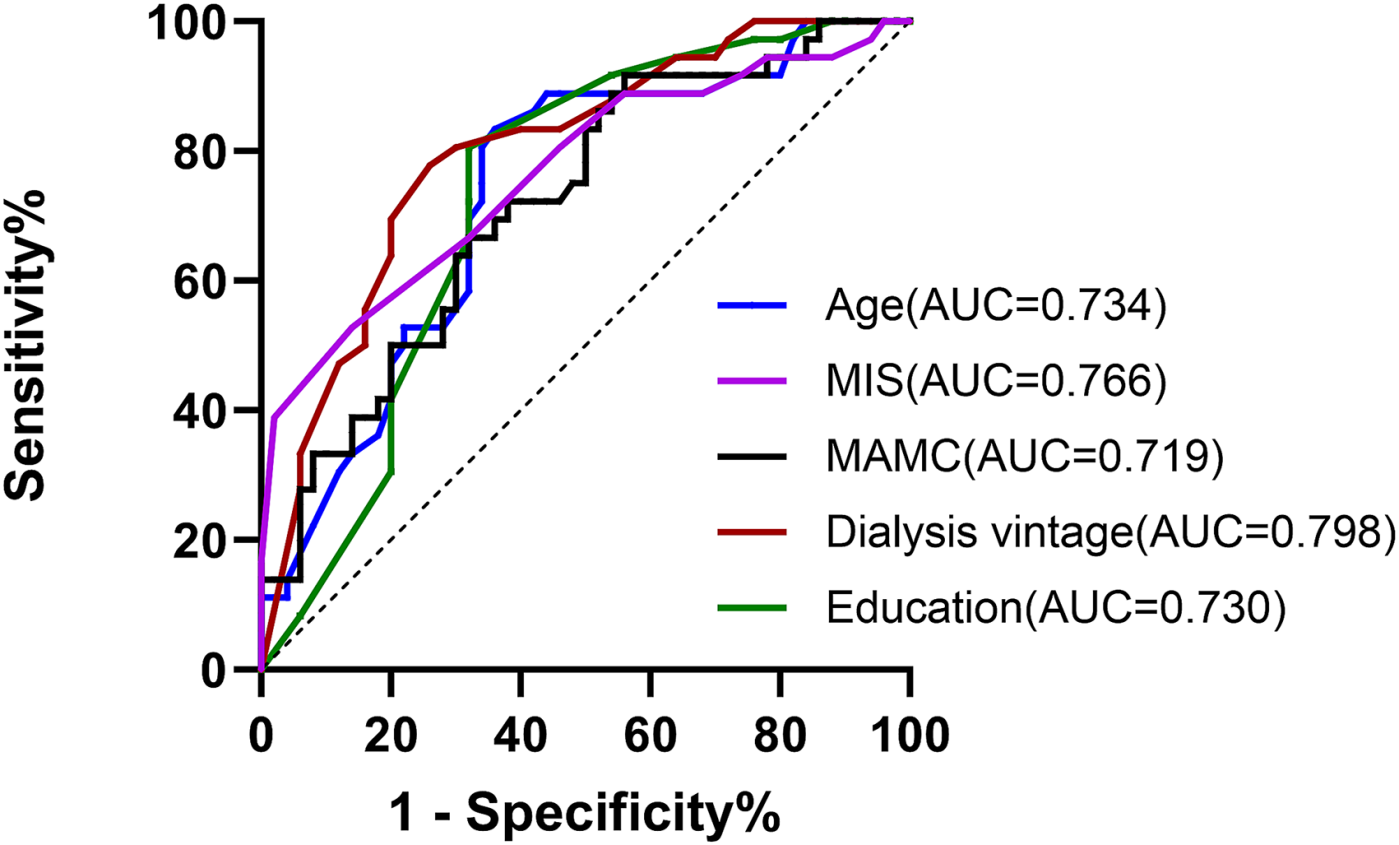
ROC curves for key variables associated with cognitive impairment. The curves demonstrate the cross-sectional discriminative ability of age, years of education, dialysis vintage, MAMC, and MIS in distinguishing patients with and without cognitive impairment. The AUC with 95% confidence intervals reflects performance within this cohort.

ROC curve analysis was performed using the five independent predictors identified above (age, years of education, dialysis vintage, MAMC, and MIS). This combined model demonstrated excellent discriminative performance (AUC = 0.942, 95% CI 0.898–0.987). This combined model substantially outperformed any single variable, with dialysis vintage showing the highest individual AUC (0.798) and MAMC demonstrating the highest specificity (91.7% at the optimal cut-off of ≤22.07 cm). At the optimal probability cut-off of 0.546, the multivariable model achieved a sensitivity of 88.0% and specificity of 88.9% (*p* < 0.001) (Figure 3 and Supplementary Table S2).

## 4 Discussion

The prevalence of CI in the MHD patients in this study was 58.1%, which is consistent with the high prevalence of cognitive impairment reported in previous studies among MHD populations (1, 2). In the multivariable logistic regression analysis, older age (adjusted OR 1.089, 95% CI 1.016–1.168, *p* = 0.017), longer dialysis vintage (adjusted OR 1.384, 95% CI 1.121–1.709, *p* = 0.002), fewer years of education (adjusted OR 0.736, 95% CI 0.587–0.923, *p* = 0.008), lower MAMC (adjusted OR 0.571, 95% CI 0.400–0.816, *p* = 0.002), and higher MIS (adjusted OR 1.249, 95% CI 1.006–1.550, *p* = 0.044) were independently associated with cognitive impairment.

PEW was significantly more prevalent in the CI group (36.0% vs. 11.1%, *p* = 0.009), and patients with PEW had lower MoCA scores. Although hs-CRP showed a significant difference and serum albumin showed a marginal difference in univariable analyses, neither remained independently associated with cognitive impairment in the final multivariable model. These findings indicate that the integrated Malnutrition–Inflammation Score (MIS), which combines laboratory and clinical parameters, provides a more reliable and comprehensive assessment of the malnutrition-inflammation axis than isolated markers such as hs-CRP. Consequently, muscle wasting (captured by MAMC) and the broader malnutrition–inflammation complex syndrome (MICS, as captured by MIS) may influence cognitive function through partly distinct pathways that are more reliably reflected by these cumulative clinical tools than by isolated acute-phase or biochemical markers alone.

Two distinct but interconnected pathways may underlie these findings. First, long-term protein depletion and muscle loss (reflected by lower MAMC) lead to essential amino acid deficiency, impairing neuronal substrate supply and neurotransmitter synthesis. In addition, reduced skeletal muscle mass decreases secretion of myokines such as irisin and brain-derived neurotrophic factor (BDNF), thereby impairing hippocampal neurogenesis, synaptic plasticity, and learning/memory capacity. Existing evidence indicates that BDNF mediates the link between muscle mass and cognitive function, and this relationship may be further amplified in the uremic environment (16–18).

Second, PEW is frequently accompanied by persistent systemic inflammation and microglial activation (reflected by higher MIS), which promote the release of pro-inflammatory cytokines such as IL-6 and IL-18 (19–22), and induce oxidative stress (23). These processes result in increased reactive oxygen species, endothelial dysfunction, and increased blood–brain barrier permeability, ultimately exacerbating neuroinflammation (24, 25).

MAMC and MIS demonstrated more robust associations with cognitive impairment than single biochemical markers or the composite PEW definition. This is likely because these tools better capture the chronic, cumulative nature of the malnutrition-inflammation axis in dialysis patients, whereas hs-CRP primarily reflects recent or acute inflammation and albumin is susceptible to short-term confounders such as volume status and recent intake.

The association between CI and PEW is likely bidirectional. Cognitive deficits can undermine adherence, appetite, and self-care, setting the stage for worsening nutrition and muscle loss. PEW-related metabolic disturbances, chronic inflammation, and progressive muscle wasting may exacerbate brain vulnerability in the uremic milieu through impaired neurotransmitter synthesis, increased neuroinflammation, and disrupted muscle-brain signaling (19–21, 23). However, due to the cross-sectional design of this study, we cannot determine the temporal sequence or causal direction between PEW and CI, which represents a key limitation. Although Mendelian randomization studies in general populations support bidirectional links between sarcopenia-like traits and cognition (26–28), these findings may not be directly extrapolatable to MHD patients, given the unique uremic-inflammatory environment and hemodynamic fluctuations during dialysis. Future prospective longitudinal studies with repeated measurements of muscle mass and cognitive function, together with Mendelian randomization analyses specifically conducted in patients with end-stage renal disease, are warranted to clarify the directionality and potential causality of this association and to determine whether preserving muscle mass can protect cognitive function in this high-risk population.

From a clinical perspective, the findings of this study suggest that maintaining a higher MAMC and achieving a lower MIS represent important modifiable targets for preserving cognitive function in patients undergoing MHD.

The study by Amicone et al. (29) offers valuable insight into early intervention strategies. In patients with non-dialysis CKD stages 3–5, a plant-based low-protein diet maintained stable muscle mass while demonstrating a trend toward improved muscle strength. It also significantly reduced inflammatory markers such as fibrinogen and effectively prevented the progression of malnutrition-inflammation complex syndrome (MICS), as reflected by stable MIS scores.

Integrating these observations with our findings of independent associations between lower MAMC, higher MIS, and cognitive impairment, we propose that early implementation of nutritional strategies focused on “muscle preservation and inflammation control” may help sustain myokine secretion, mitigate long-term neuroinflammation, and provide strategic benefits for subsequent cognitive protection. Consequently, the management of MICS should be viewed as a continuous clinical process (continuum) beginning in the pre-dialysis phase rather than being confined to the dialysis period.

Although the cross-sectional design of this study precludes causal inference regarding the relationships between MAMC, MIS, and cognitive impairment, these results underscore the importance of monitoring muscle mass and inflammatory status across CKD disease stages for potential cognitive benefits in high-risk populations. In terms of clinical applicability, the multivariable discrimination model constructed in this study demonstrated excellent discriminative performance (AUC = 0.942, 95% CI 0.898–0.987), outperforming traditional single factors. Specifically, MAMC at the optimal cut-off of ≤22.07 cm showed high specificity (91.7%), making it useful for ruling out cognitive impairment, whereas MIS at ≥3.5 exhibited high sensitivity (86.0%), facilitating early identification of high-risk individuals. These findings indicate that incorporating MAMC and MIS into routine nutritional-cognitive screening holds strong clinical utility.

Several limitations deserve mention. First, as a cross-sectional study, this work cannot establish causality or the temporal sequence between PEW and cognitive impairment—a bidirectional possibility that has been discussed in detail in the Discussion section. Second, the single-center sample (*n* = 86) limits generalizability, particularly to centers with different case-mix or dialysis practices. Although the events-per-variable ratio of 10.0 meets the recommended threshold of 10, the modest sample size still entails a risk of model overfitting; thus, our findings require validation in larger independent cohorts. Third, even with education-adjusted MoCA scores, residual cultural or educational bias cannot be fully excluded. Fourth, we did not assess important psychological confounders such as depression and sleep disturbances, which are known to influence both nutritional parameters and cognitive function (30). This may have resulted in residual confounding of the observed associations. Fifth, we used dialysis vintage rather than total CKD duration as the exposure variable. Dialysis vintage may not fully reflect cumulative uremic toxin exposure, as some patients may have initiated renal replacement therapy at different stages of CKD progression. Finally, absence of myokine profiling or brain imaging left mechanistic insights indirect.

In summary, lower MAMC and higher MIS emerged as the most robust nutritional correlates of CI in this MHD population, outperforming both conventional biomarkers and the full PEW syndrome definition. These results highlight a potentially important muscle-brain link in uremia and underscore the need for longitudinal studies to disentangle directionality and test whether preserving muscle mass can meaningfully protect cognition in this high-risk group.

## Data Availability

The original contributions presented in the study are included in the article/Supplementary material, further inquiries can be directed to the corresponding author.

## References

[1] Mobushar JA Shahzad A Mumtaz B Naeem I Zafar AA Jamil MI . Prevalence and pattern of cognitive impairment in patients on maintenance hemodialysis. Cureus. (2025) 17:e84649. doi: 10.7759/cureus.84649 40546548 PMC12182912

[2] Golenia A Żołek N Olejnik P Żebrowski P Małyszko J. Patterns of cognitive impairment in hemodialysis patients and related factors including depression and anxiety. J Clin Med. (2023) 12:3119. doi: 10.3390/jcm12093119, 37176560 PMC10179667

[3] Chien CW Lin YC Huang SK Chen PE Tung TH. A population-based study of the association between hemodialysis and cognitive impairment. Asia Pac Psychiatry. (2020) 12:e12404. doi: 10.1111/appy.12404 32715665

[4] Guo Y Tian R Ye P Li X Li G Lu F . Cognitive domain impairment and all-cause mortality in older patients undergoing hemodialysis. Front Endocrinol (Lausanne). (2022) 13:828162. doi: 10.3389/fendo.2022.828162, 35418951 PMC8995766

[5] van Zwieten A Wong G Ruospo M Palmer SC Teixeira-Pinto A Barulli MR . Associations of cognitive function and education level with all-cause mortality in adults on hemodialysis: findings from the COGNITIVE-HD study. Am J Kidney Dis. (2019) 74:452–62. doi: 10.1053/j.ajkd.2019.03.424, 31160141

[6] Takagi K Takahashi H Miura T Yamagiwa K Kawase K Muramatsu-Maekawa Y . Prognostic value of the controlling nutritional status (CONUT) score in patients at dialysis initiation. Nutrients. (2022) 14:2317. doi: 10.3390/nu14112317, 35684116 PMC9182995

[7] Lee H Kim K Ahn J Lee DR Lee JH Hwang SD. Association of nutritional status with osteoporosis, sarcopenia, and cognitive impairment in patients on hemodialysis. Asia Pac J Clin Nutr. (2020) 29:712–23. doi: 10.6133/apjcn.202012_29(4).0006, 33377365

[8] Yang X Quan Y Wu E Jiang Y Song Q Li Y . The association of cognition with protein energy wasting and synaptic transmission in chronic kidney disease. Semin Dial. (2023) 36:326–36. doi: 10.1111/sdi.13146, 36864620

[9] Rotondi S Tartaglione L Pasquali M Ceravolo MJ Mitterhofer AP Noce A . Association between cognitive impairment and malnutrition in hemodialysis patients: two sides of the same coin. Nutrients. (2023) 15:813. doi: 10.3390/nu15040813, 36839171 PMC9964006

[10] Fouque D Kalantar-Zadeh K Kopple J Cano N Chauveau P Cuppari L . A proposed nomenclature and diagnostic criteria for protein-energy wasting in acute and chronic kidney disease. Kidney Int. (2008) 73:391–8. doi: 10.1038/sj.ki.5002585, 18094682

[11] Obi Y Qader H Kovesdy CP Kalantar-Zadeh K. Latest consensus and update on protein-energy wasting in chronic kidney disease. Curr Opin Clin Nutr Metab Care. (2015) 18:254–62. doi: 10.1097/MCO.0000000000000171, 25807354 PMC4506466

[12] Carrero JJ Thomas F Nagy K Arogundade F Avesani CM Chan M . Global prevalence of protein-energy wasting in kidney disease: a meta-analysis of contemporary observational studies from the International Society of Renal Nutrition and Metabolism. J Ren Nutr. (2018) 28:380–92. doi: 10.1053/j.jrn.2018.08.006, 30348259

[13] Nasreddine ZS Phillips NA Bédirian V Charbonneau S Whitehead V Collin I . The Montreal cognitive assessment, MoCA: a brief screening tool for mild cognitive impairment. J Am Geriatr Soc. (2005) 53:695–9. doi: 10.1111/j.1532-5415.2005.53221.x, 15817019

[14] van Zwieten A Wong G Ruospo M Palmer SC Barulli MR Iurillo A . Prevalence and patterns of cognitive impairment in adult hemodialysis patients: the COGNITIVE-HD study. Nephrol Dial Transplant. (2018) 33:1197–206. doi: 10.1093/ndt/gfx314, 29186522

[15] Peduzzi P Concato J Kemper E Holford TR Feinstein AR. A simulation study of the number of events per variable in logistic regression analysis. J Clin Epidemiol. (1996) 49:1373–9. doi: 10.1016/S0895-4356(96)00236-3, 8970487

[16] Uchida K Sugimoto T Tange C Nishita Y Shimokata H Saji N . Association between reduction of muscle mass and faster declines in global cognition among older people: a 4-year prospective cohort study. J Nutr Health Aging. (2023) 27:932–9. doi: 10.1007/s12603-023-2007-9, 37997712 PMC12876671

[17] Pedersen BK. Physical activity and muscle-brain crosstalk. Nat Rev Endocrinol. (2019) 15:383–92. doi: 10.1038/s41574-019-0174-x, 30837717

[18] Kim S Choi JY Moon S Park DH Kwak HB Kang JH. Roles of myokines in exercise-induced improvement of neuropsychiatric function. Pflugers Arch. (2019) 471:491–505. doi: 10.1007/s00424-019-02253-8, 30627775

[19] Ebert T Neytchev O Witasp A Kublickiene K Stenvinkel P Shiels PG. Inflammation and oxidative stress in chronic kidney disease and dialysis patients. Antioxid Redox Signal. (2021) 35:1426–48. doi: 10.1089/ars.2020.8184, 34006115

[20] Chou ML Babamale AO Walker TL Cognasse F Blum D Burnouf T. Blood-brain crosstalk: the roles of neutrophils, platelets, and neutrophil extracellular traps in neuropathologies. Trends Neurosci. (2023) 46:764–79. doi: 10.1016/j.tins.2023.06.005, 37500363

[21] Tsai MT Ou SM Chen HY Tseng WC Lee KH Yang CY . Relationship between circulating Galectin-3, systemic inflammation, and protein-energy wasting in chronic hemodialysis patients. Nutrients. (2021) 13:2803. doi: 10.3390/nu13082803, 34444962 PMC8398098

[22] Liu Q Sanai N Jin WN La Cava A Van Kaer L Shi FD. Neural stem cells sustain natural killer cells that dictate recovery from brain inflammation. Nat Neurosci. (2016) 19:243–52. doi: 10.1038/nn.4211, 26752157 PMC5336309

[23] Duni A Liakopoulos V Roumeliotis S Peschos D Dounousi E. Oxidative stress in the pathogenesis and evolution of chronic kidney disease: untangling Ariadne’s thread. Int J Mol Sci. (2019) 20:3711. doi: 10.3390/ijms20153711, 31362427 PMC6695865

[24] Bobot M Guedj E Resseguier N Faraut J Garrigue P Nail V . Increased blood-brain barrier permeability and cognitive impairment in patients with ESKD. Kidney Int Rep. (2024) 9:2988–95. doi: 10.1016/j.ekir.2024.07.021, 39430169 PMC11489453

[25] Assem M Lando M Grissi M Kamel S Massy ZA Chillon JM . The impact of uremic toxins on cerebrovascular and cognitive disorders. Toxins (Basel). (2018) 10:303. doi: 10.3390/toxins10070303, 30037144 PMC6071092

[26] Liu H Fan Y Liang J Hu A Chen W Wang H . A causal relationship between sarcopenia and cognitive impairment: a Mendelian randomization study. PLoS One. (2024) 19:e0309124. doi: 10.1371/journal.pone.0309124, 39240885 PMC11379137

[27] Duan L Li H Li S Shi Y Feng Y. Causal association between sarcopenia and cognitive impairment contributes to the muscle-brain axis: a bidirectional Mendelian randomization study. Geriatr Gerontol Int. (2025) 25:116–22. doi: 10.1111/ggi.15045, 39660394

[28] Zhou Y Guo X Dong S Wang C Wu L Liu W. Causal relationship between sarcopenia and cognitive performance: a bidirectional Mendelian randomization study. Medicine (Baltimore). (2025) 104:e45718. doi: 10.1097/MD.0000000000045718, 41204539 PMC12599665

[29] Amicone M Di Lauro M Rizzo M Di Lorenzo T Gigliotti G Pisani A . Plant-based low-protein diet for preventing malnutrition–inflammation complex syndrome in adults with CKD: a single-centre preliminary experience. BMC Nephrol. (2026) 27:102. doi: 10.1186/s12882-025-04729-5, 41526893 PMC12888280

[30] Guenzani D Buoli M Caldiroli L Carnevali GS Serati M Vezza C . Malnutrition and inflammation are associated with severity of depressive and cognitive symptoms of old patients affected by chronic kidney disease. J Psychosom Res. (2019) 124:109783. doi: 10.1016/j.jpsychores.2019.109783, 31443824

