# Supplementary material for: Association between protein-energy wasting and cognitive impairment in maintenance hemodialysis patients
Source: Front Nutr. 2026 Apr 29;13:1825670. doi: 10.3389/fnut.2026.1825670 (PMC13167555; doi:10.3389/fnut.2026.1825670)
Supplement: Supplementary file 1 [file Table_1.pdf]

Supplementary Material

Supplementary Table 1. Comparison of Cognitive Function Scores

	CI Group (n=50)	NCI Group (n=36)	Z	P value
MoCA Subdomains (score)				
Visuospatial/Executive	3.0 (2.0, 3.0)	4.5 (4.0, 5.0)	-6.810	<0.001
Naming	3.0 (2.0, 3.0)	3.0 (3.0, 3.0)	-3.352	<0.001
Attention	5.0 (4.0, 6.0)	6.0 (6.0, 6.0)	-5.877	<0.001
Language	2.0 (1.0, 3.0)	3.0 (3.0, 3.0)	-5.808	<0.001
Abstraction	1.0 (0.0, 1.0)	2.0 (1.0, 2.0)	-4.869	<0.001
Delayed Recall	1.0 (0.0, 2.0)	3.0 (3.0, 4.0)	-6.918	<0.001
Orientation	6.0 (5.0, 6.0)	6.0 (6.0, 6.0)	-3.647	<0.001
Trail Making Test				
TMT-A Completion Time, s	55.1 (46.5, 119.8)	40.1 (31.6, 48.0)	4.924	<0.001
TMT-B Completion Time, s	84.0 (70.1, 119.5)	43.5 (36.2, 50.8)	6.404	<0.001
TMT-B Sequencing Errors, n	4.0 (2.0, 6.0)	1.0 (0.0, 2.0)	4.927	<0.001
TMT-B Correct Connections, n	20.0 (18.0, 22.3)	23.0 (22.0, 24.0)	-4.812	<0.001

CI: cognitive impairment; NCI: non-cognitive impairment; MoCA: Montreal Cognitive Assessment; TMT: Trail Making Test.

Supplementary Table 2. Cross-sectional discriminative performance of factors associated with cognitive impairment

	Optimal Cut-off	AUC (95% CI)	Sensitivity	Specificity	P value
Age	>53 years	0.734 (0.627–0.841)	0.640	0.833	<0.001
Education	≤9.5 years	0.730 (0.623–0.837)	0.680	0.806	<0.001
Dialysis vintage	>4.75 years	0.798 (0.704–0.893)	0.740	0.778	<0.001
MAMC	≤22.07 cm	0.719 (0.611–0.827)	0.440	0.917	0.001
MIS	≥3.5	0.766(0.661–0.870)	0.860	0.528	<0.001
Multivariable model	Predicted probability ≥ 0.546	0.942(0.898–0.987)	0.880	0.889	<0.001

Note: The multivariable model was constructed using the five independent variables identified in logistic regression analysis.

Abbreviations: AUC = area under the curve; MAMC = mid-arm muscle circumference; MIS = Malnutrition–Inflammation Score.
